# Dietary Inflammatory Index and Its Relationship with Cervical Carcinogenesis Risk in Korean Women: A Case-Control Study

**DOI:** 10.3390/cancers11081108

**Published:** 2019-08-03

**Authors:** Sundara Raj Sreeja, Hyun Yi Lee, Minji Kwon, Nitin Shivappa, James R. Hebert, Mi Kyung Kim

**Affiliations:** 1 Cancer Epidemiology Branch, Division of Cancer Epidemiology and Prevention, National Cancer Center, Madu-dong, Ilsan-dong-gu, Goyang-si, Gyeonggi-do 10408, Korea; 2 Cancer Prevention and Control Program, University of South Carolina, Columbia, SC 29208, USA; 3 Department of Epidemiology and Biostatistics, Arnold School of Public Health, University of South Carolina, Columbia, SC 29208, USA; 4 Connecting Health Innovations Columbia, Columbia, SC 29201, USA

**Keywords:** cervical cancer, dietary inflammatory index, inflammatory biomarkers, cervical intraepithelial neoplasia

## Abstract

Several studies have reported that diet’s inflammatory potential is related to chronic diseases such as cancer, but its relationship with cervical cancer risk has not been studied yet. The aim of this study was to investigate the association between Dietary Inflammatory Index (DII^®^) and cervical cancer risk among Korean women. This study consisted of 764 cases with cervical intraepithelial neoplasia (CIN)1, 2, 3, or cervical cancer, and 729 controls from six gynecologic oncology clinics in South Korea. The DII was computed using a validated semiquantitative Food Frequency Questionnaire (FFQ). Odds ratios and 95% CI were calculated using multinomial logistic regression. Higher DII scores were associated with higher cervical carcinogenesis risk. A significant association was observed between the DII and risk among CIN2/3 [Odds Ratio (OR) = 3.14; 95% Confidence Intervals (CI) = 1.57–6.29] and cervical cancer patients (OR = 1.98; 95% CI = 1.01–3.88). Among Human Papilloma Virus (HPV)-positive women, a significant association was found between DII and cervical carcinoma risk with CIN2/3 (OR = 5.65; 95% CI = 1.38–23.2). Moreover, women with CIN2/3 and cervical cancer showed a significant association with proinflammatory diet in people without of physical activity (OR = 3.79; 95% CI = 1.81–7.93). These findings suggest that high intake of proinflammatory diets is associated with increased risk of cervical carcinogenesis among women with CIN2/3. Further evaluation in future studies to confirm this association is warranted.

## 1. Introduction

Cervical cancer is a life-threatening gynecologic malignancy arising in the lining of the cervix in women, particularly in less developed and developing countries [1]. It is estimated that within 10–20 years, incidence and mortality rates of cervical carcinoma will increase all over the world [2]. According to the global cancer statistics of the World Cancer Research Fund, 569,847 new cancer cases of cervical cancer were diagnosed in 2018, accounting for 6.9% of the total number of new cases diagnosed in 2018 worldwide [3]. Among Korean women, cervical cancer is the seventh most common cancer and third leading cause of cancer-associated death [4]. Cervical malignancy is preventable only if detected at its early stage by the presence of precancerous lesions [1,4]. Human Papilloma Virus (HPV) is regarded a causal factor for cervical carcinogenesis [5,6]. Other risk factors for cervical cancer include poor dietary habits, smoking, weak immune system, overweight, usage of oral contraceptives, and family history [7,8,9].

Previous studies reveal that diet is associated with both cancer and inflammation [10,11,12,13]. Dietary supplementation with antioxidants, including minerals, vitamins, and phenolic compounds, maintains a desirable antioxidative balance by reducing oxidative processes and inflammation [11]. Inflammatory markers, such as C-reactive protein, Tumor Necrosis Factor (TNF), and Interleukin 6, which are associated with chronic diseases, are modulated by diet [12]. Increased consumption of polyphenols from plant products with antioxidant properties has been reducing the risk of cancer [13].

The Dietary Inflammatory Index (DII^®^) describes a dietary pattern that classifies an individual’s diet on a continuum ranging from anti-inflammatory to proinflammatory [14]. Proinflammatory foods include red meat, fried foods, high-fat dairy products, and refined grains [15]. By contrast, anti-inflammatory foods such as green vegetables, fruits, olive oil, and whole grains are associated with lower levels of inflammation [16]. High DII scores indicate a diet’s increased inflammatory potential, and these have been associated with increased risk of malignancy [17,18].

Several studies suggest that there is a direct association between inflammatory dietary patterns and cervical cancer [19,20]. According to the European Prospective Investigation into Cancer and Nutrition Study, high consumption of anti-inflammatory nutrients such as fiber, carotenoids, antioxidants, and polyphenols is associated with reduced risk of cervical intraepithelial neoplasia (CIN) and cervical carcinoma among healthy women [21]. Vitamin E is considered to be a powerful antioxidant that protects cells from DNA damage and mutagenesis, thereby preventing tumors in the cervix [22]. In cervical cancer patients, only trace amounts of vitamin C are found, which results in a lack of protection against increased oxidative stress due to the consumption of proinflammatory diets rich in protein, trans fat and carbohydrate [23].

Numerous epidemiological studies have been conducted to analyze the association between DII scores and various cancers (e.g., pancreatic, bladder, breast, lung, and ovarian) [15,24,25,26]. From all of those studies, it was evident that proinflammatory diet showed positive significant association with various cancers. The present study is the first to focus on the putative association of DII and cervical cancer. According to an Italian case-control study, there was a strong and significant association between proinflammatory diet and bladder cancer risk [17]. Similarly, in a New Jersey case-control study, a significant association was observed between proinflammatory diet and ovarian cancer risk among postmenopausal women [26]. In an Iranian case-control study, it was reported that women who consumed more proinflammatory diet were at higher risk of breast cancer, especially among premenopausal women [24]. In a Prostate, Lung, Colorectal, and Ovarian cohort study, no significant associations were observed between inflammatory potential of diet and pancreatic cancer risk, but it did show significant associations in the follow-up years [15]. The main aim of the current study was to analyze the association between DII and cervical carcinogenesis among Korean women. Its hypothesis is that individuals with higher DII scores are at elevated risk of developing cervical carcinoma.

## 2. Results

The distribution of the demographic characteristics of the patients with cervical intraepithelial neoplasia (CIN) grades 1, 2, or 3, and cervical cancer, along with those of the controls, are presented in Table 1. The CINs and cervical cancer cases were more likely to be married, to have completed high school, to have a medium income and to be physically active. A higher prevalence of physical activity was observed among women with CIN1 and cervical cancer cases when compared with the controls and the CIN2/3 case groups. A higher prevalence of drinking was observed among women with CINs and cervical cancer when compared with the controls. The majority of the pre- and peri-menopausal women were CINs when compared with the cervical cancer patients.

The distribution of the selected food parameters of the Dietary Inflammatory Index (DII^®^) among the 764 cases and 729 controls is shown in Table 2. Statistically significant associations were found for anti-inflammatory foods and nutrients such as carotene, monounsaturated fatty acids (MUFA), polyunsaturated fatty acids (PUFA), fiber, garlic, ginger, n-3 fatty acids, n-6 fatty acids, niacin, onion, pepper, turmeric, and vitamins A, B1, B2, C, D, and E among the CINs and cervical cancer cases. Significant associations were observed for proinflammatory dietary components such as energy, saturated fat, total fat, iron, protein, and vitamin B12. Significant heterogeneity was observed for carotene, MUFA, PUFA, saturated fat, trans-fat, total fat, fiber, garlic, ginger, iron, n-3 fatty acids, n-6 fatty acids, pepper and vitamins A, B2, B6, B12, C, and E.

The odds ratios of cervical cancer risk and the corresponding 95% confidence intervals according to quintiles of DII among the cases and controls are presented in Table 3. A statistically significant association was observed between DII and the risk of CIN2/3 and cervical cancer. When the analysis was carried out with the DII expressed as quintiles, CIN2/3 women had the highest risk by intake of proinflammatory diet (OR = 3.14; 95% CI = 1.57–6.29; *p*-trend = 0.0005). High consumption of proinflammatory dietary parameters was not associated with increased risk among women with CIN1 (OR = 0.86; 95% CI = 0.48–1.55). In the fully adjusted model, cervical cancer women in the highest quintile had a statistically significant increased risk of cervical cancer in comparison with the lowest quintile (OR = 1.98; 95% CI = 1.01–3.88). When the DII was used as a continuous measure, significant results were obtained among CIN2/3 cases but not for CIN1 (OR = 1.01; 95% CI = 0.91–1.10) or cervical cancer (OR = 1.12 95% CI = 1.00–1.24) cases. Among the CIN2/3 patients, one unit increase in DII was associated with a 23% increase in the odds of cervical cancer risk (OR = 1.23; 95% CI = 1.10–1.37).

Table 4 provides the odds ratios of cervical cancer risk and DII as stratified by HPV status and physical activity. When the DII was expressed as quintiles, statistically significant results were obtained among HPV-positive women with CIN 2/3 (OR = 5.65; 95% CI = 1.38–23.2), and with a significant trend (*p*-trend = 0.03), despite the insignificant results obtained for HPV-positive CIN1 cases (OR = 0.93; 95% CI = 0.34–2.53) and HPV-positive cervical cancer patients (OR = 0.76; 95% CI = 0.15–3.77) with nonsignificant trends. The *p*-value for interaction was not significant for DII and HPV infection. When the DII was expressed as a continuous measure, in HPV-positive women, similarly significant associations were found between DII and cervical cancer risk with CIN 2/3 (OR = 1.27; 95% CI = 1.02–1.57). Among the HPV-negative women, when the DII was expressed as quintiles, significant associations were found between proinflammatory diet and risk among CIN2/3 cases (OR = 2.36; 95% CI = 1.04–5.35). When the DII was used as a continuous measure, significant associations were observed among HPV-negative women consuming the most proinflammatory diet (OR = 1.20; 95% CI = 1.06–1.36). No associations were observed between DII and cervical cancer risk among HPV-negative women with CIN1 and cervical cancer.

The association between the DII and risk was significant among CIN 2/3 (OR = 3.79; 95% CI = 1.81–7.93) (*p*-trend ≤ 0.0001) and cervical cancer cases (OR = 2.11; 95% CI = 1.04–4.28) (*p*-trend = 0.008) with no physical activity. Similarly, the results obtained for using DII as a continuous variable in relation to cervical cancer risk showed significant associations for women with CIN 2/3 (OR = 1.28; 95% CI = 1.14–1.43) and cervical cancer (OR = 1.14, 95% CI = 1.02–1.73) with no physical activity. Among the participants performing physical activity, no associations were found between proinflammatory diet and risks of CIN grades 1, 2, and 3 and cervical cancer. The *p*-value for interaction was not significant for the DII and physical activity.

The odds ratios of CINs and cervical cancer for quintiles of anti-inflammatory food and nutrient parameters are presented in Table 5 and Table S1. Significantly higher intakes of ginger (OR = 1.72; 95% CI = 1.11–2.67) and vitamin B1 (OR = 1.74; 95% CI = 1.09–2.77) intakes were observed among CIN1 cases compared with the CIN 2/3 and cervical cancer groups. Moreover, a statistically significant association was found between the DII and cervical cancer risk among the CIN 2/3 cases by consumption of tea (OR = 1.66; 95% CI = 1.08–2.56) (p-value=0.02). Carotene, fiber, garlic, and vitamins A and C were found to be negatively associated with CIN grades 1, 2, and 3 and cervical cancer cases.

## 3. Discussion

This case-control study which included 764 cases and 729 controls aimed to determine the association between the inflammatory potential of diet (as indicated by DII score) and CIN risk. We observed that higher DII scores, as indicative of more proinflammatory diets, were associated with increased risk of CIN. Particularly, women with higher DII scores were at higher risk of CIN2/3. HPV-positive women showed a strong relationship between DII dietary patterns and cervical cancer risk by the presence of cervical intraepithelial lesions. Moreover, physical activity was significantly associated with DII and risk among CIN2/3 and cervical cancer. Thus, the results confirmed the study hypothesis, which is to say, that higher DII scores are associated with increased risk of cervical carcinogenesis.

Although this is the first attempt to examine the association between DII and cervical carcinoma risk, several investigations into the association between dietary patterns and CINs, which leads to cervical cancer, revealed similar results. A cross-sectional study conducted in Italy reported that Western dietary patterns, which include large amounts of trans fat, sodium, protein, cholesterol, saturated fat, and carbohydrate, were significantly associated with high incidence rates of cervical cancer [20]. Similarly, two studies reported that unhealthy diets that are rich in fat, processed meat, starchy foods, and sweets increased the risk of developing cervical intraepithelial neoplasia (CIN) among women [27,28]. In both of these studies, it was evident that dietary patterns were strongly associated with CIN, a precursor lesion for development of cervical cancer. Moreover, it was suggested that cancer-protective micronutrients such as folate; vitamins A, C, B2, B6, and E; and carotene, all factors that decrease DII scores, should be included in diets [29,30].

The current study found a statistically significant association between DII and cervical carcinogenesis risk among women with CIN2/3 and cervical cancer. However, the results were not significant for women with CIN1. There are no previous reports on any association of DII with cervical cancer risk, and moreover, there are few reports of evidence for any significant association between DII and inflammatory biomarkers of cervical cancer and their differences in disease progression [31]. According to a Brazilian cross-sectional study, increasing concentrations of serum α- and γ-tocopherols and higher dietary intakes of dark-green and deep-yellow vegetables and fruits were associated with decreased risk of CIN3 [32]. Similarly, another cross-sectional study found that consumption of a more proinflammatory diet was associated with increased levels of inflammatory biomarkers such as TNF-α, IL-1,2, IFN-γ and VCAM among healthy individuals and a resultantly increased risk of CINs [33]. In CIN 2/3 and cervical carcinoma women, impaired cell-mediated immune response has been observed by with deregulation of immune-system mediators such as cytokines, adhesion molecules and their receptors. However, in CIN 1, immune response induces the regression of HPV infection in most women in spite of immune invasion and downregulation of the immune system [34]. These differences in immune-system functioning result in increased risk of cervical cancer among women with CIN 2/3. So, further studies are needed to analyze the association between DII and cervical cancer risk among women with CINs.

A statistically significant association between DII and cervical cancer risk was revealed among HPV-positive women with CIN 2/3. An earlier study reported that changes in dietary patterns for high(er) intakes of green tea and vegetables were considered as a protective factor for CINs and cervical cancer [35]. According to a Brazilian cohort study based on the data from the Ludwig-McGill HPV Natural History Study revealed that HPV-positive women with CIN 2/3 were significantly associated with low intakes of dietary nutrients such as lutein, folate, β-cryptoxanthin and vitamin C [36]. Moreover, vitamin C, vitamin E, and other dietary constituents that maintain normal methylation level inhibit DNA adduct formation by modulating inflammatory response and suppressing the expression of HPV oncogenes, which results in reducing the risk of cervical cancer [20,37]. These are the important determinants of the severity of cervical cancer progression in women with CIN 2/3. So, further investigations have to be conducted to more fully understand the association between DII and cervical cancer risk with regard to high risk of HPV infection.

Another important finding of the present study was that women not engaging in regular physical activity had increased risk of CIN2/3 and cervical cancer by consumption of a proinflammatory diet. A previous study reported that physical activity was inversely associated with CIN 2/3 and cervical cancer [38]. Physical activity acts as an immune modulator and induces the activity of macrophages, natural killer cells, and neutrophils. Additionally, irregular physical activity increases homocysteine levels, which in turn increase the risk of CIN 2/3 among women [39,40].

The DII score was developed to assess the inflammatory potential of diet. Two studies have obtained data on 29 and 31 food parameters for DII development, respectively [17,26]. In the present study, however, 33 food parameters were available, and these additional parameters provided for more accurate DII scores. Overall, the DII score was high for women subjects consuming more proinflammatory food and nutrient parameters in their diets.

The major strength of the present study is the fact that it is the first to explore the association between DII and cervical cancer risk among Korean women. Moreover, the study includes a large sample size with a valid FFQused to assess dietary data. Another strength is the use of DII scores to access the inflammatory potential of diets, because this approach takes into account both proinflammatory and anti-inflammatory food parameters that characterize human diet. Furthermore, significant results obtained in this study underline the importance of consuming an anti-inflammatory diet to prevent cervical carcinoma risk. Notwithstanding such strengths, this study should be considered in view of its limitations. As a case-control design for a Korean population, there was a possibility of selection and information bias based on dietary habits and health concerns. Another limitation is that only 33 of the 45 food parameters were taken for DII development. So, the remaining 12 food parameters, if also utilized, could have influenced the results. Additionally, in this study, the levels of inflammatory biomarkers were not measured, because disease progression severity was evaluated by the presence of well-known histopathological parameters.

## 4. Materials and Methods

### 4.1. Subject Recruitment

This hospital-based case-control study was conducted at The National Cancer Center (NCC) of Korea from March 2006 to 2010. It was approved by the ethics committees of NCC and of each of the pertinent centers (IRB: NCC2016-0147). The study included 764 cases and 729 controls. These women had been selected randomly from the gynecologic oncology clinics of six university hospitals in Korea. Among the 764 cases, 319 were diagnosed with CIN1, 216 with CIN2/3, and 229 with CX CAN. Details on the study’s design criteria are available in our previous paper [41]. The cases were patients with pathologically confirmed cervical carcinoma as diagnosed six months prior to the interview. The controls were patients admitted to the same network of hospitals for diseases other than cervical cancer. Both the cases and controls were selected by a simple random sampling technique. The cases were selected based on certain factors such as histopathologically confirmed cervical cancer, free of conditions such as hysterectomy and oophorectomy, and willingness to participate in the study. Demographic characteristics were gathered using a structured questionnaire that included questions related to smoking, drinking, physical activity, menopausal status, and oral contraceptive usage. Informed consent was obtained from all of the participants prior to the study.

### 4.2. Calculation of DII

Food Frequency Questionnaire (FFQ)-derived dietary data were used to calculate the DII scores for all of the participants. The FFQ consists of dietary data on 95 food and beverage items and queries on frequency and portion size for each item [42]. We recorded usual dietary factors with details on food intakes over the year prior to enrollment for each of the participants, including the frequency of consumption and portion sizes. The frequency of each food item consumed was classified into nine categories: almost never, once per month, 2–3 times per month, 1–2 times per week, 3–4 times per week, 5–6 times per week, once per day, twice per day, and three times per day. The standard portion size of each food item was determined using the mean amount, the standard value or the natural unit as referenced in the Korea Ministry of Health and Welfare portion size booklet [43]. Portion size in the SQFFQ was divided into three categories: small (half the medium portion), medium and large (1.5 times or greater the medium portion). The medium intake was determined by the mean amount for the study subjects. The usual food intakes derived from the FQQ were determined by multiplying the frequency of consumption by the daily portion size for each food group. Nutrient intake for each food item was calculated using the Diet Analysis program (version 4.0) for nutrients.

A complete description of the DII is available elsewhere [14]. Briefly, in the updated version of the DII, a total of 1943 articles were peer-reviewed and scored. Scoring for each food parameter was based on its inflammatory potential on six inflammatory biomarkers including C-reactive protein, IL-1β, IL-4, IL-6, IL-10, and tumor necrosis factor-alpha. The dietary information on each participant were first linked to food consumption data sets from 11 countries around the world to estimate the average and standard deviation for each of the 45 food parameters. Z scores were calculated by subtracting the global standard average from the amount reported and dividing by its standard deviation. These Z scores were converted to centered proportions in order to minimize the right-skewing effect. Each obtained value was multiplied by the corresponding food parameter effect score. All of the food parameter-specific DII scores were summed to obtain the overall DII score. High-positive scores indicated a more proinflammatory diet, whereas high-negative scores indicated a more anti-inflammatory diet. In this study, the following 33 of the possible 45 food parameters were considered for DII development; cholesterol, carotene, energy, carbohydrate, MUFA, PUFA, saturated fat, trans fat, total fat, fiber, garlic, ginger, iron, magnesium, selenium, zinc, n-3 fatty acids, n-6 fatty acids, niacin, onion, pepper, protein, tea, turmeric, folic acid, and vitamins A, B1, B2, B6, B12, C, D, and E.

### 4.3. Measurement of Covariates

During the collection of the participants’ socio-demographic characteristics, age, BMI, and energy intake were considered as continuous variables. Marital status, education level, income, smoking, drinking, physical activity, pregnancy, oral contraceptive usage, and menopausal status were considered as categorical variables. Marital status was further classified into single, married, and divorced. Education level was classified as middle school or below, high school or below and college or above. Similarly, menopausal status was classified into pre- or peri-menopause (presence of monthly menstrual cycle) and post-menopause (absence of monthly menstrual cycle). Smoking (no: nonsmoker; yes: smoker), drinking (no: nondrinker; yes: drinker), physical activity (no: irregular; yes: regular), and oral contraceptive usage participants (no: never; yes: current user) were classified accordingly. The number of missing data for categorical variables were as follows, marital status (*n* = 1), educational status (*n* = 3), income (*n* = 2), smoking (*n* = 2), alcohol consumption (*n* = 3), physical activity (*n* = 6), pregnancy (*n* = 5), oral contraception (*n* = 4), and menopausal status (*n* = 3).

### 4.4. Statistical Analyses

The DII quintiles were defined by the distribution of DII scores in the control group. The medians and 25th and 75th percentile values were calculated for the continuous variables, and frequencies with percentages were calculated for the categorical variables. The Jonckheere-Terpstra and Mantel-Haenszel tests were used to compare the categorical and continuous variables, respectively. The distribution of DII components was estimated among the groups using the Kruskal-Wallis and Jonckheere-Terpstra tests. The odds ratios (OR) and corresponding 95% confidence intervals (CI) were estimated using multinomial logistic regression. The minimally adjusted model was adjusted for age and energy intake; the fully adjusted model was additionally adjusted for marital status, education, smoking, alcohol consumption, physical activity, pregnancy history, use of oral contraception, menopausal status, and BMI. Stratified analyses were carried out by HPV status and physical activity. To investigate the DII components and the risk of cervical cancer progression, the intake foods were adjusted for energy density. P for trends were calculated using quintiles of DII. *p*-values less than 0.05 were considered statistically significant. All of the statistical analyses were performed with SAS^®^ 9.3 (SAS Institute Inc, Cary, NC, USA).

## 5. Conclusions

The results of this study support a significant association between proinflammatory diet and cervical carcinogenesis risk, especially in women with CIN2/3. Also, a significant association was observed in HPV-positive women with CIN2/3. Intake of more anti-inflammatory dietary factors, such as omega-3 fatty acids, plant-based foods rich in fiber, beta carotenes, and phytochemicals, with reduced intake of proinflammatory factors such as fried foods or processed foods rich in saturated fat or trans fatty acids, might be a strategy for mitigating the risk of some types of cervical cancer. Further evaluation in future studies will help to improve the present understanding of the association between DII and cervical cancer risk and of healthy diets that regulate the level of inflammatory biomarkers; thereby, cervical carcinogenesis risk will be reduced and public health will be promoted.

## Figures and Tables

**Table 1:** Distribution of demographic characteristics of study subjects.

Characteristics		Controls (*n* = 729)	Cases (*n* = 764)			*p*-Value ^a^
			CIN1 (*n* = 319)	CIN2/3 (*n* = 216)	CX CAN (*n* = 229)	
DII		0.5 (−1.3, 2.0)	0.1 (−1.6, 1.6)	1.1 (−0.7, 2.5)	1.5 (−0.6, 3.0)	0.0003
Age (years)		43 (35, 51)	38 (31, 47)	39 (32, 47)	48 (42, 58)	0.12
BMI (kg/m^2^)		21.9 (20.2, 24.1)	21.4 (19.7, 23.5)	21.4 (19.5, 23.7)	23 (20.8, 25.3)	0.30
Energy (kcal/day)		1850 (1512, 2178)	1969 (1631, 2296)	1826 (1585, 2238)	1767 (1493, 2104)	0.73
Marital status						
	Single	78 (10.7)	70 (21.9)	29 (13.4)	10 (4.5)	<0.0001
	Married	568 (78.1)	213 (66.8)	156 (72.2)	160 (69.9)	
	Divorced	81 (11.2)	36 (11.3)	31 (14.4)	59 (25.6)	
Education level						
	≤Middle School	155 (21.4)	54 (16.9)	58 (27.0)	115 (50.2)	<0.0001
	High School	31 (42.9)	138 (43.3)	95 (44.2)	87 (38.0)	
	≥College	260 (35.7)	127 (39.8)	62 (28.8)	27 (11.8)	
Income (10,000 won) ^b^						
	Less than 200	194 (26.7)	93 (29.2)	70 (32.4)	128 (55.8)	<0.0001
	200–500	417 (57.2)	179 (56.3)	130 (60.2)	94 (41.1)	
	More than 500	117 (16.1)	46 (14.5)	16 (7.4)	7 (3.1)	
Smoking						
	No	631 (86.6)	260 (81.5)	178 (83.2)	194 (84.7)	0.28
	Yes	98 (13.4)	59 (18.5)	36 (16.8)	35 (15.3)	
Alcohol consumption						
	No	285 (39.2)	77 (24.2)	68 (31.5)	106 (46.3)	0.50
	Yes	442 (60.8)	241 (75.8)	148 (68.5)	123 (53.7)	
Physical activity ^c^						
	No	595 (82.1)	264 (82.8)	194 (89.8)	207 (91.2)	0.0001
	Yes	130 (17.9)	55 (17.2)	22 (10.2)	20 (8.8)	
Pregnancy						
	No	127 (17.5)	93 (29.3)	48 (22.3)	13 (5.7)	0.01
	Yes	601 (82.5)	225 (70.7)	167 (77.7)	214 (94.3)	
Oral contraception						
	No	607 (83.4)	263 (82.7)	167 (77.7)	182 (79.8)	0.08
	Yes	121 (16.6)	55 (17.3)	48 (22.3)	46 (20.2)	
Menopausal status						
	Pre/Peri	481 (66.1)	255 (80.2)	171 (79.2)	87 (38.2)	<0.0001
	Post	247 (33.9)	63 (19.8)	45 (20.8)	141 (61.8)	

Data are presented as medians (25th, 75th) in continuous variables and as *n* (%) in categorical variables. DII, dietary inflammatory index; CIN, cervical intraepithelial neoplasia; BMI, body-mass index; CX CAN, cervical cancer. ^a^ *p* values were calculated by Jonckheere–Terpstra test for continuous variables and Mantel–Haenszel test for categorical variables. ^b^ What is the average monthly household income (total income of all living together, including yourself)? ^c^ Did you have any intense physical activity such as walking, jogging or running during the last 7 days?

**Table 2:** Distribution of selected food parameters and nutrients of Dietary Inflammatory Index (DII) among 764 cases and 729 controls.

Components	Controls (*n* = 729)	Cases			*p* ^a^	*p* ^b^
		CIN1 (*n* = 319)	CIN2/3 (*n* = 216)	CX CAN (*n* = 229)		
**Pro–Inflammatory**						
Energy (kcal)	1850 (1512–2178)	1969 (1631–2296)	1826 (1586–2238)	1766 (1493–2104)	0.002	0.73
Carbohydrate (g)	303 (246–353)	314 (255–373)	310 (257357)	301 (247–351)	0.13	0.69
Protein (g)	65.7 (54.2–83.3)	70.1 (57.3–88.6)	66.9 (54.8–84.4)	62.5 (47.5–81.2)	0.003	0.26
Total fat (g)	38.2 (28.3–49.5)	41.1 (30.5–54.8)	37.4 (27.9–52.5)	33.3 (23–47.1)	<0.0001	0.02
Saturated fat(g)	7.3 (4.9–10.3)	7.7 (5.3–10.8)	7.5 (4.6–10.3)	6.4 (3.9–9.2)	0.0004	0.05
Trans fat (g)	0.1 (0–0.1)	0.1 (0–0.2)	0.1 (0–0.2)	0.1 (0–0.2)	<0.0001	0.002
Cholesterol (mg)	149 (99.9–224)	170 (108–248)	162 (104–248)	126.9 (75.5–198)	<0.0001	0.16
Iron (mg)	12.9 (10.3–16.7)	13.6 (10.8–16.9)	12.2 (9.8–15.7)	11.9 (9.1–15.4)	0.004	0.04
Vitamin B12	4.1 (2.6–5.8)	4.1 (2.7–6.1)	4 (2.5–5.9)	3.5 (2.1–5)	0.002	0.02
**Anti–Inflammatory**						
n3 fatty acids (g)	1.7 (1.2–2.4)	1.7 (1.2–2.4)	1.6 (1.2–2.3)	1.6 (1–2.2)	0.03	0.02
n6 fatty acids (g)	9.6 (7.3–1)	9.8 (7.4–13.4)	8.9 (6.9–12.8)	8.4 (5.7–12.1)	0.002	0.002
MUFA (g)	8.9 (6.1–12.4)	9.5 (6.4–12.9)	9 (5.9–12.2)	7.4 (4.6–11.1)	<0.0001	0.006
Vitamin A (RE)	934 (698–1290)	1024 (763–1423)	805 (597–1136)	764 (558–1106)	<0.0001	<0.0001
Carotene (μg)	5061 (3810–7187)	5544 (3993–7618)	4250 (3119–6021)	4207 (3092–6028)	<0.0001	<0.0001
Vitamin B1(mg)	1.1 (0.9–1.4)	1.2 (1–1.5)	1.1 (0.9–1.4)	1.1 (0.8–1.3)	<0.0001	0.08
Vitamin B2 (mg)	1.2 (1–1.5)	1.3 (1–1.6)	1.2 (0.9–1.5)	1.1 (0.8–1.4)	<0.0001	0.02
Niacin (mg)	16.8 (13.7–21.2)	18 (14.4–22.1)	16.7 (14–20.9)	15.6 (12–20.3)	0.001	0.13
Vitamin B6	1.6 (1.3–2)	1.7 (1.4–2.1)	1.5 (1.2–1.9)	1.5 (1.1–2)	0.001	0.04
PUFA (g)	7.1 (4.9–10.1)	7.4 (4.9–10.2)	6.8 (4.7–10.2)	6.3 (4–8.8)	0.001	0.003
Folic acid (μg)	365 (274–486)	372 (286–485)	345 (260–504)	333 (239–482)	0.05	0.09
Vitamin C (mg)	141 (106–203)	156 (112–214)	122 (86.5–168)	116 (89.3–164)	<0.0001	<0.0001
Vitamin D (μg)	9.4 (6.3–14.2)	11.1 (7.9–16.5)	9.8 (6.3–13.5)	7.7 (4.5–13.1)	<0.0001	0.12
Vitamin E (mg)	7.4 (5.6–10.1)	7.8 (5.9–10.3)	6.7 (5.3–9.1)	6.3 (4.6–9)	<0.0001	0.001
Fiber (g)	8.4 (6.6–11.3)	8.9 (6.8–11.8)	7.6 (5.9–9.9)	7.5 (5.9–10.1)	<0.0001	0.001
Magnesium (mg)	200. (165–242)	202.4 (165–246)	197 (160–238)	189 (152–237)	0.08	0.06
Selenium (μg)	41.7 (33.3–50.4)	42.7 (32.1–52.1)	43.7 (34.3–50.9)	44 (34.3–52.9)	0.23	0.05
Zinc (mg)	6.6 (5.5–7.9)	6.8 (5.7–8.2)	6.8 (5.7–8)	6.6 (5.1–7.8)	0.21	0.82
Garlic (g)	6.2 (4.6–8.5)	7 (5.1–9.4)	5.9 (4.3–8.1)	5.6 (4.0–8.3)	<0.0001	0.04
Ginger (g)	0.7 (0.5–1.1)	0.8 (0.6–1.2)	0.6 (0.4–0.9)	0.6 (0.4–1.0)	<0.0001	0.02
Onion (g)	12.2 (7.8–17.8)	14.4 (9.7–19.9)	12 (8.5–19.3)	10.2 (6.3–16.8)	<0.0001	0.28
Pepper (g)	6.5 (4.41–9)	6.4 (4.5–9.9)	5.1 (3.6–7.8)	5.1 (3.6–7.8)	<0.0001	<0.0001
Tea (g)	16.9 (0–78.7)	16.9 (0–78.7)	21.4 (0–100)	16.9 (0–80.2)	0.12	0.21
Turmeric (mg)	0.1 (0–0.2)	0.1 (0–0.2)	0.1 (0–0.2)	0.03 (0–0.1)	<0.0001	0.10

MUFA: monounsaturated fatty acids; PUFA: polyunsaturated fatty acids. ^a^ *p*-values were calculated by Kruskal–Wallis test to identify significant differences among the groups. ^b^ *p*-values were calculated by Jonckheere–Terpstra test to identify trends in the continuous variables.

**Table 3:** Odds ratios (ORs) of cervical cancer risk and corresponding 95% confidence intervals (CI) according to quintiles of Dietary Inflammatory Index (DII) among 764 cases and 729 controls.

Variables	Quintiles of Dietary Inflammatory Index					P for Trend ^c^	DII Continuous
	Q1 (Anti-Inflammatory)	Q2	Q3	Q4	Q5 (Proinflammatory)		
Minimally adjusted model ^a^							
CIN1	Ref	0.96 (0.62–1.49)	1.16 (0.73–1.83)	0.89 (0.54–1.49)	0.80 (0.45–1.42)	0.46	0.99 (0.90–1.08)
CIN2/3	Ref	1.34 (0.76–2.38)	1.94 (1.08–3.50)	2.70 (1.46–5.01)	3.70 (1.90–7.22)	<0.0001	1.27 (1.15–1.41)
CX CAN	Ref	0.96 (0.55–1.68)	1.01 (0.56–1.84)	1.25 (0.68–2.31)	2.63 (1.40–4.95)	0.0003	1.18 (1.07–1.30)
Fully adjusted model ^b^							
CIN1	Ref	0.97 (0.62–1.51)	1.23 (0.77–1.97)	0.96 (0.57–1.62)	0.86 (0.48–1.55)	0.67	1.01 (0.91–1.10)
CIN2/3	Ref	1.41 (0.78–2.54)	1.89 (1.03–3.48)	2.45 (1.28–4.66)	3.14 (1.57–6.29)	0.0005	1.23 (1.10–1.37)
CX CAN	Ref	0.93 (0.52–1.67)	0.92 (0.49–1.73)	0.99 (0.52–1.89)	1.98 (1.01–3.88)	0.02	1.12 (1.00–1.24)

^a^ Minimally adjusted model: adjusted for age (continuous) and energy (continuous). ^b^ Fully adjusted model: adjusted for age (continuous), energy (continuous), marriage (single, married, divorced), education level (middle school, high school, college), alcohol consumption (yes, no), physical activity (yes, no), pregnancy (yes, no), oral contraceptive use (yes, no), menopausal status (pre- or peri-menopause, post-menopause) and BMI (continuous). ^c^ Categorical DII scores by quintiles were used to determine p for trends.

**Table 4:** Multivariate odds ratios (ORs) of cervical cancer risk and corresponding 95% confidence intervals (CIs) for quintiles of Dietary Inflammatory Index (DII) among 764 cases and 729 controls by selected strata.

Variables	Quintiles of Dietary Inflammatory Index					P for Trends ^a^	DII Continuous	P for Interaction ^b^
	Q1 (Anti-Inflammatory)	Q2	Q3	Q4	Q5 (Proinflammatory)			
HPV infection								
HPV positive								
CIN1	Ref	0.85 (0.42–1.73) ^a^	1.19 (0.54–2.63)	1.10 (0.45–2.66)	0.93 (0.34–2.53)	0.8646	1.00 (0.85–1.17)	0.57
CIN2/3	Ref	2.67 (0.85–8.41)	3.07 (0.87–10.8)	3.89 (1.02–14.8)	5.65 (1.38–23.2)	0.0251	1.27 (1.02–1.57)	
CX CAN	Ref	0.48 (0.13–1.78)	0.93 (0.24–3.65)	0.05 (0.00–0.54)	0.76 (0.15–3.77)	0.5736	0.94 (0.72–1.23)	
HPV negative								
CIN1	Ref	0.99 (0.55–1.81)	1.27 (0.69–2.34)	0.95 (0.48–1.87)	0.83 (0.39–1.77)	0.5607	1.00 (0.89–1.13)	
CIN2/3	Ref	1.08 (0.53–2.20)	1.50 (0.73–3.06)	2.07 (0.97–4.40)	2.36 (1.04–5.35)	0.0122	1.20 (1.06–1.36)	
CX CAN	Ref	1.27 (0.69–2.34)	0.82 (0.39–1.69)	1.23 (0.59–2.55)	1.98 (0.91–4.30)	0.0249	1.12 (0.99–1.27)	
Physical activity ^d^								
No activity								
CIN1	Ref	0.76 (0.46–1.27)	1.28 (0.76–2.16)	1.03 (0.58–1.83)	0.95 (0.48–1.88)	0.8287	1.02 (0.92–1.14)	0.41
CIN2/3	Ref	1.33 (0.70–2.52)	1.90 (0.98–3.66)	2.60 (1.31–5.19)	3.79 (1.81–7.93)	<0.0001	1.28 (1.14–1.43)	
CX CAN	Ref	0.83 (0.45–1.55)	0.81 (0.41–1.58)	0.95 (0.48–1.88)	2.11 (1.04–4.28)	0.0084	1.14 (1.02–1.73)	
Activity								
CIN1	Ref	1.94 (0.72–5.25)	0.88 (0.27–2.88)	0.51 (0.13–2.04)	0.58 (0.12–2.71)	0.1451	0.92 (0.72–1.17)	
CIN2/3	Ref	1.98 (0.35–11.2)	3.07 (0.42–22.4)	4.61 (0.50–42.7)	0.75 (0.04–14.9)	0.7718	1.04 (0.69–1.57)	
CX CAN	Ref	2.19 (0.29–16.7)	2.59 (0.30–22.5)	4.10 (0.37–45.0)	1.39 (0.10–19.1)	0.8626	0.94 (0.63–1.42)	

^a^ Categorical DII scores by quintiles were used to determine p for trends. ^b^ The multiplicative terms (categorical DII × HPV infection, categorical DII × physical activity) for the interaction. ^c^ Adjusted for age (continuous), energy (continuous), marriage (single, married, divorced), education level (middle school, high school, college), drinking (yes, no), physical activity (yes, no), pregnancy (yes, no), oral contraceptive use (yes, no), menopausal status (pre- or peri-menopause, post-menopause) and BMI (continuous). ^d^ Did you have any intense physical activity such as walking, jogging or running during the last 7 days?

**Table 5:** Odds ratios (ORs) of CINs and cervical cancer for quintiles of anti-inflammatory food and nutrient parameters.

DII Component	Anti-Inflammatory Food and Nutrient Parameters					P for Trends ^a^
	Q1	Q2	Q3	Q4	Q5	
Vitamin A						
CIN1	Ref	0.95 (0.61–1.48) ^b^	1.12 (0.73–1.73)	0.87 (0.55–1.38)	1.39 (0.91–2.13)	0.19
CIN2/3	Ref	0.83 (0.54–1.29)	0.65 (0.40–1.03)	0.36 (0.21–0.63)	0.40 (0.23–0.67)	<0.0001
CX CAN	Ref	0.75 (0.48–1.18)	0.42 (0.26–0.69)	0.30 (0.17–0.52)	0.65 (0.40–1.03)	0.001
β-carotene						
CIN1	Ref	1.01 (0.65–1.56)	1.03 (0.66–1.59)	0.98 (0.63–1.53)	1.41 (0.92–2.15)	0.16
CIN2/3	Ref	0.76 (0.49–1.18)	0.64 (0.40–1.01)	0.35 (0.20–0.61)	0.41 (0.24–0.69)	<0.0001
CX CAN	Ref	0.96 (0.62–1.50)	0.41 (0.25–0.69)	0.35 (0.20–0.61)	0.66 (0.41–1.06)	0.0013
Vitamin B1						
CIN1	Ref	1.74 (1.10–2.74)	1.40 (0.87–2.24)	1.36 (0.85–2.19)	1.74 (1.09–2.77)	0.14
CIN2/3	Ref	0.87 (0.54–1.40)	1.00 (0.63–1.59)	0.66 (0.40–1.10)	0.46 (0.26–0.81)	0.006
CX CAN	Ref	0.66 (0.42–1.06)	0.71 (0.44–1.13)	0.52 (0.31–0.88)	0.65 (0.39–1.07)	0.03
Vitamin C						
CIN1	Ref	0.88 (0.57–1.36)	0.95 (0.62–1.46)	0.86 (0.55–1.34)	1.29 (0.85–1.95)	0.29
CIN2/3	Ref	1.15 (0.75–1.76)	0.57 (0.35–0.94)	0.49 (0.29–0.83)	0.35 (0.20–0.62)	<0.0001
CX CAN	Ref	0.96 (0.62–1.49)	0.47 (0.28–0.78)	0.31 (0.18–0.55)	0.57 (0.35–0.92)	<0.0001
Fiber						
CIN1	Ref	0.81 (0.52–1.24)	0.99 (0.65–1.50)	0.83 (0.53–1.29)	1.21 (0.79–1.85)	0.42
CIN2/3	Ref	0.94 (0.61–1.45)	0.69 (0.44–1.10)	0.36 (0.20–0.63)	0.40 (0.23–0.69)	<0.0001
CX CAN	Ref	0.97 (0.61–1.56)	0.68 (0.42–1.12)	0.42 (0.24–0.72)	0.62 (0.37–1.02)	0.003
Garlic						
CIN1	Ref	1.10 (0.70–1.72)	1.41 (0.90–2.19)	1.15 (0.73–1.80)	1.48 (0.95–2.31)	0.10
CIN2/3	Ref	0.97 (0.61–1.53)	0.72 (0.44–1.19)	0.83 (0.52–1.34)	0.51 (0.30–0.88)	0.02
CX CAN	Ref	1.05 (0.66–1.68)	0.74 (0.45–1.22)	0.58 (0.34–0.98)	0.95 (0.59–1.55)	0.24
Ginger						
CIN1	Ref	1.17 (0.74–1.86)	1.61 (1.04–2.50)	0.98 (0.61–1.57)	1.72 (1.11–2.67)	0.06
CIN2/3	Ref	1.13 (0.72–1.76)	0.89 (0.55–1.42)	0.46 (0.27–0.79)	0.53 (0.31–0.91)	0.0006
CX CAN	Ref	1.08 (0.69–1.70)	0.52 (0.31–0.88)	0.34 (0.19–0.61)	0.88 (0.55–1.41)	0.03
Tea						
CIN1	Ref	0.86 (0.48–1.56)	1.38 (0.95–2.00)	1.10 (0.74–1.63)	0.78 (0.51–1.18)	0.55
CIN2/3	Ref	0.70 (0.34–1.48)	1.18 (0.75–1.87)	1.12 (0.69–1.80)	1.66 (1.08–2.56)	0.02
CX CAN	Ref	0.71 (0.35–1.45)	0.95 (0.60–1.50)	1.15 (0.72–1.83)	1.33 (0.85–2.06)	0.18

All of the food and nutrient parameters were adjusted for energy by the density method. ^a^ Categorical food parameter scores by quintiles were used to determine p for trends. ^b^ Fully adjusted model: adjusted for age (continuous), marriage (single, married, divorced), education level (middle school, high school, college), drinking (yes, no), pregnancy (yes, no), oral contraceptive use (yes, no), menopausal status (pre- or peri-menopause, post-menopause) and BMI (continuous).

## Supplementary Materials

The following are available online at https://www.mdpi.com/2072-6694/11/8/1108/s1, Table S1: Odds ratios (ORs) of CINs and cervical cancer for quintiles of 24 anti-inflammatory food and nutrient parameters.
