# Supplementary material for: Dietary Inflammatory Index and Its Relationship with Cervical Carcinogenesis Risk in Korean Women: A Case-Control Study
Source: Cancers (Basel). 2019 Aug 3;11(8):1108. doi: 10.3390/cancers11081108 (PMC6721526; doi:10.3390/cancers11081108)
Supplement: Supplementary file 1 [file cancers-11-01108-s001.pdf]

Supplementary Material: Dietary Inflammatory Index and Its Relationship with Cervical Carcinogenesis Risk in Korean Women: A Case-Control Study

Sundara Raj Sreeja, Hyun Yi Lee, Minji Kwon, Nitin Shivappa, James R. Hebert and Mi Kyung Kim

Table S1. Odds ratios (ORs) of CINs and cervical cancer for quintiles of 24 anti-inflammatory food and nutrient parameters.

DII Component		Anti-Inflammatory Food and Nutrient Parameters					*p* for Trends [a]
		Q1	Q2	Q3	Q4	Q5	
n3 fatty acids							
	CIN1	ref	1.11 (0.73–1.67)	0.87 (0.57–1.33)	0.74 (0.48–1.15)	0.70 (0.45–1.09)	0.02
	CIN2/3	ref	0.95 (0.58–1.55)	1.27 (0.8–2.020)	0.75 (0.45–1.27)	0.77 (0.46–1.29)	0.23
	CC	ref	0.91 (0.56–1.47)	0.98 (0.61–1.58)	0.85 (0.52–1.41)	0.91 (0.55–1.50)	0.63
n6 fatty acids							
	CIN1	ref	1.06 (0.69–1.64)	1.12 (0.73–1.73)	0.83 (0.53–1.30)	0.63 (0.39–1.01)	0.03
	CIN2/3	ref	1.37 (0.86–2.18)	1.03 (0.63–1.68)	0.59 (0.34–1.02)	0.66 (0.38–1.15)	0.01
	CC	ref	1.16 (0.74–1.83)	0.97 (0.60–1.57)	0.79 (0.47–1.33)	0.74 (0.43–1.27)	0.10
MUFA							
	CIN1	ref	0.86 (0.55–1.36)	1.19 (0.77–1.85)	0.88 (0.56–1.38)	0.66 (0.41–1.07)	0.13
	CIN2/3	ref	1.09 (0.66–1.80)	1.15 (0.69–1.91)	1.07 (0.64–1.79)	0.74 (0.42–1.30)	0.33
	CC	ref	1.06 (0.68–1.67)	0.93 (0.57–1.52)	0.98 (0.59–1.65)	0.65 (0.36–1.15)	0.16
Vitamin A							
	CIN1	ref	0.95 (0.61–1.48)	1.12 (0.73–1.73)	0.87 (0.55–1.38)	1.39 (0.91–2.13)	0.19
	CIN2/3	ref	0.83 (0.54–1.29)	0.65 (0.40–1.03)	0.36 (0.21–0.63)	0.40 (0.23–0.67)	<.0001
	CC	ref	0.75 (0.48–1.18)	0.42 (0.26–0.69)	0.30 (0.17–0.52)	0.65 (0.40–1.03)	0.001
Carotene							
	CIN1	ref	1.01 (0.65–1.56)	1.03 (0.66–1.59)	0.98 (0.63–1.53)	1.41 (0.92–2.15)	0.16
	CIN2/3	ref	0.76 (0.49–1.18)	0.64 (0.40–1.01)	0.35 (0.20–0.61)	0.41 (0.24–0.69)	<.0001
	CC	ref	0.96 (0.62–1.50)	0.41 (0.25–0.69)	0.35 (0.20–0.61)	0.66 (0.41–1.06)	0.001
Vitamin B1							
	CIN1	ref	1.74 (1.10–2.74)	1.40 (0.87–2.24)	1.36 (0.85–2.19)	1.74 (1.09–2.77)	0.14
	CIN2/3	ref	0.87 (0.54–1.40)	1.00 (0.63–1.59)	0.66 (0.40–1.10)	0.46 (0.26–0.81)	0.01
	CC	ref	0.66 (0.42–1.06)	0.71 (0.44–1.13)	0.52 (0.31–0.88)	0.65 (0.39–1.07)	0.03
Vitamin B2							
	CIN1	ref	1.33 (0.84–2.08)	1.28 (0.81–2.02)	1.32 (0.84–2.08)	1.12 (0.71–1.79)	0.76
	CIN2/3	ref	0.94 (0.59–1.51)	0.93 (0.58–1.51)	0.76 (0.46–1.26)	0.49 (0.28–0.85)	0.01
	CC	ref	0.69 (0.43–1.11)	0.90 (0.56–1.46)	0.85 (0.51–1.41)	0.78 (0.47–1.30)	0.51
Niacin							

	CIN1	ref	0.83 (0.53–1.29)	1.19 (0.78–1.81)	0.79 (0.51–1.25)	0.98 (0.63–1.54)	0.89
	CIN2/3	ref	1.11 (0.68–1.83)	1.44 (0.89–2.34)	1.18 (0.71–1.97)	0.65 (0.36–1.18)	0.37
	CC	ref	1.03 (0.64–1.67)	1.39 (0.87–2.22)	1.00 (0.6–1.67)	0.77 (0.45–1.33)	0.48
Vitamin B6							
	CIN1	ref	1.1 (0.73–1.68)	0.89 (0.58–1.38)	1.05 (0.68–1.61)	0.82 (0.52–1.29)	0.38
	CIN2/3	ref	0.94 (0.6–1.48)	1.02 (0.64–1.62)	0.59 (0.35–1)	0.55 (0.32–0.95)	0.01
	CC	ref	0.72 (0.45–1.16)	0.89 (0.55–1.44)	0.76 (0.47–1.26)	0.74 (0.44–1.23)	0.29
PUFA							
	CIN1	ref	0.92 (0.60–1.42)	0.99 (0.65–1.53)	0.81 (0.52–1.27)	0.47 (0.29–0.77)	0.01
	CIN2/3	ref	1.14 (0.70–1.84)	1.05 (0.64–1.72)	0.82 (0.49–1.38)	0.68 (0.39–1.20)	0.08
	CC	ref	1.17 (0.74–1.86)	1.14 (0.70–1.84)	1.08 (0.65–1.80)	0.64 (0.36–1.15)	0.21
Folic acid							
	CIN1	ref	1.14 (0.75–1.76)	1.19 (0.78–1.82)	1.19 (0.78–1.81)	0.75 (0.47–1.18)	0.37
	CIN2/3	ref	0.88 (0.55–1.42)	0.80 (0.49–1.31)	0.71 (0.43–1.17)	0.87 (0.54–1.42)	0.36
	CC	ref	0.62 (0.38–1.01)	0.74 (0.45–1.22)	0.79 (0.48–1.29)	1.05 (0.65–1.69)	0.70
Vitamin C							
	CIN1	ref	0.88 (0.57–1.36)	0.95 (0.62–1.46)	0.86 (0.55–1.34)	1.29 (0.85–1.95)	0.29
	CIN2/3	ref	1.15 (0.75–1.76)	0.57 (0.35–0.94)	0.49 (0.29–0.83)	0.35 (0.20–0.62)	<.0001
	CC	ref	0.96 (0.62–1.49)	0.47 (0.28–0.78)	0.31 (0.18–0.55)	0.57 (0.35–0.92)	<.0001
Vitamin D							
	CIN1	ref	1.39 (0.85–2.26)	1.69 (1.05–2.71)	1.68 (1.04–2.71)	1.65 (1.02–2.66)	0.04
	CIN2/3	ref	1.81 (1.08–3.03)	1.24 (0.72–2.12)	1.62 (0.95–2.74)	0.91 (0.51–1.63)	0.57
	CC	ref	0.96 (0.6–1.52)	0.69 (0.41–1.14)	0.81 (0.49–1.34)	0.76 (0.46–1.27)	0.17
Vitamin E							
	CIN1	ref	1.13 (0.72–1.76)	1.2 (0.77–1.87)	1.33 (0.86–2.07)	0.86 (0.54–1.39)	0.87
	CIN2/3	ref	0.99 (0.62–1.58)	0.98 (0.61–1.57)	0.57 (0.33–0.97)	0.64 (0.37–1.09)	0.01
	CC	ref	0.75 (0.47–1.21)	1.21 (0.76–1.93)	0.97 (0.59–1.6)	0.57 (0.32–1.01)	0.23
Fiber							
	CIN1	ref	0.81 (0.52–1.24)	0.99 (0.65–1.50)	0.83 (0.53–1.29)	1.21 (0.79–1.85)	0.42
	CIN2/3	ref	0.94 (0.61–1.45)	0.69 (0.44–1.10)	0.36 (0.20–0.63)	0.40 (0.23–0.69)	<.0001
	CC	ref	0.97 (0.61–1.56)	0.68 (0.42–1.12)	0.42 (0.24–0.72)	0.62 (0.37–1.02)	0.003
Magnesium							
	CIN1	ref	0.82 (0.54–1.23)	0.80 (0.53–1.20)	0.64 (0.41–0.98)	0.61 (0.39–0.95)	0.013
	CIN2/3	ref	1.02 (0.65–1.59)	0.64 (0.39–1.05)	0.66 (0.4–1.10)	0.57 (0.34–0.98)	0.01
	CC	ref	1.11 (0.68–1.81)	0.74 (0.44–1.25)	0.79 (0.47–1.34)	1.14 (0.69–1.88)	0.88
Selenium							
	CIN1	ref	1.06 (0.7–1.60)	0.98 (0.64–1.51)	1.09 (0.70–1.70)	1.01 (0.64–1.61)	0.93
	CIN2/3	ref	1.20 (0.72–1.99)	0.89 (0.51–1.53)	1.59 (0.95–2.65)	1.20 (0.70–2.05)	0.30
	CC	ref	1.39 (0.74–2.6)	1.76 (0.96–3.23)	1.99 (1.11–3.58)	1.56 (0.85–2.85)	0.11
Zinc							

	CIN1	ref	1.00 (0.66–1.50)	1.02 (0.68–1.54)	1.02 (0.67–1.56)	0.54 (0.34–0.87)	0.05
	CIN2/3	ref	1.05 (0.64–1.70)	0.91 (0.55–1.50)	1.11 (0.67–1.82)	0.91 (0.55–1.52)	0.82
	CC	ref	0.84 (0.49–1.44)	0.91 (0.53–1.54)	1.17 (0.71–1.95)	1.29 (0.78–2.14)	0.12
Garlic							
	CIN1	ref	1.10 (0.70–1.72)	1.41 (0.90–2.19)	1.15 (0.73–1.80)	1.48 (0.95–2.31)	0.10
	CIN2/3	ref	0.97 (0.61–1.53)	0.72 (0.44–1.19)	0.83 (0.52–1.34)	0.51 (0.30–0.88)	0.02
	CC	ref	1.05 (0.66–1.68)	0.74 (0.45–1.22)	0.58 (0.34–0.98)	0.95 (0.59–1.55)	0.24
Ginger							
	CIN1	ref	1.17 (0.74–1.86)	1.61 (1.04–2.50)	0.98 (0.61–1.57)	1.72 (1.11–2.67)	0.06
	CIN2/3	ref	1.13 (0.72–1.76)	0.89 (0.55–1.42)	0.46 (0.27–0.79)	0.53 (0.31–0.91)	0.001
	CC	ref	1.08 (0.69–1.70)	0.52 (0.31–0.88)	0.34 (0.19–0.61)	0.88 (0.55–1.41)	0.03
Onion							
	CIN1	ref	1.77 (1.08–2.91)	2.07 (1.26–3.39)	1.46 (0.87–2.45)	1.83 (1.1–3.05)	0.15
	CIN2/3	ref	1.48 (0.88–2.47)	1.12 (0.65–1.94)	1.33 (0.77–2.30)	1.26 (0.72–2.2)	0.64
	CC	ref	0.90 (0.56–1.45)	1.12 (0.69–1.83)	1.39 (0.84–2.30)	0.98 (0.56–1.71)	0.54
Pepper							
	CIN1	ref	1.30 (0.85–1.97)	0.96 (0.62–1.49)	1.07 (0.69–1.66)	1.05 (0.68–1.64)	0.82
	CIN2/3	ref	1.22 (0.80–1.88)	0.52 (0.31–0.87)	0.51 (0.30–0.87)	0.50 (0.29–0.86)	<.0001
	CC	ref	0.84 (0.53–1.34)	0.79 (0.49–1.27)	0.54 (0.32–0.92)	0.53 (0.31–0.89)	0.004
Tea							
	CIN1	ref	0.86 (0.48–1.56)	1.38 (0.95–2.00)	1.10 (0.74–1.63)	0.78 (0.51–1.18)	0.55
	CIN2/3	ref	0.70 (0.34–1.48)	1.18 (0.75–1.87)	1.12 (0.69–1.80)	1.66 (1.08–2.56)	0.02
	CC	ref	0.71 (0.35–1.45)	0.95 (0.60–1.50)	1.15 (0.72–1.83)	1.33 (0.85–2.06)	0.18
Turmeric							
	CIN1	ref	1.49 (0.92–2.43)	1.22 (0.73–2.02)	1.62 (0.99–2.64)	2.17 (1.34–3.51)	0.002
	CIN2/3	ref	1.55 (0.93–2.60)	1.04 (0.59–1.81)	1.12 (0.64–1.94)	1.58 (0.92–2.70)	0.37
	CC	ref	1.02 (0.65–1.60)	0.84 (0.52–1.37)	0.65 (0.38–1.11)	0.96 (0.56–1.63)	0.33

All DII components were adjusted to energy density (DII component/energy). [a] Full-adjusted model: adjusted for age (continuous), marriage (single, married, divorced), education level (middle school, high school, college), drinking (yes, no), pregnancy (yes, no), oral contraceptive use (yes, no), menopausal status (pre or peri menopause, post menopause) and BMI (continuous).

© 2019 by the authors. Licensee MDPI, Basel, Switzerland. This article is an open access article distributed under the terms and conditions of the Creative Commons Attribution (CC BY) license (http://creativecommons.org/licenses/by/4.0/).
